# The importance of the family situation to understand the role of anorexia symptoms

**DOI:** 10.1192/j.eurpsy.2021.1870

**Published:** 2021-08-13

**Authors:** P. Del Sol Calderon, A. Izquierdo, M. García Moreno

**Affiliations:** 1 Psychiatry, Hospital Universitario Puerta de Hierro, Majadahonda, Spain; 2 Psychiatry, HOSPITAL UNIVERSITARIO PUERTA DE HIERRO MAJADAHONDA, MADRID, Spain

**Keywords:** eating disorder family dynamics

## Abstract

**Introduction:**

The objective of this poster is to show the importance of understanding the situation of the patient’s family in order to know the development and role that eating symptoms are occupying both in the patient and in the different members that make up the family

**Objectives:**

Highlight the triggering and sustaining factors of a case of anorexia nervosa

**Methods:**

Case Report

**Results:**

Patient is a 14-year-old woman who begins to develop excessive concern about her body image initiating eating behaviors in the form of high restriction and counting of calories from food. Also she explains that she began to compulsively perform more than two hours a day of sports in order to lose weight Family genogram: she is an only daughter, whose parents have been separated for 4 years. Parents recognize conflictive relationship. The patient recognizes a very close relationship with her mother. When she talks about her relationship with her father, she explains how she felt very close to her father when she was young but that after the separation her father moved away. She describes that his father rebuilt his life a year ago and that he recently informed her that he is going to be a father again. She recognizes intense feelings of abandonment from her father. She acknowledges that the sense of control starting with anorexia initially helped her to feel confident about herself.

**Conclusions:**

Understanding the origin of the symptoms and their function is essential for a better management of cases of anorexia

**Disclosure:**

No significant relationships.

